# PDBCharges: Quantum-Mechanical Partial Atomic Charges for PDB Structures

**DOI:** 10.1093/nar/gkaf401

**Published:** 2025-05-10

**Authors:** Ondřej Schindler, Tomáš Svoboda, Adrián Rošinec, Tomáš Raček, Gabriela Bučeková, Dominik Tichý, Karel Berka, Radka Svobodová

**Affiliations:** CEITEC—Central European Institute of Technology, Masaryk University, 625 00 Brno, Czech Republic; National Centre for Biomolecular Research, Faculty of Science, Masaryk University, 625 00 Brno, Czech Republic; CEITEC—Central European Institute of Technology, Masaryk University, 625 00 Brno, Czech Republic; National Centre for Biomolecular Research, Faculty of Science, Masaryk University, 625 00 Brno, Czech Republic; Institute of Computer Science, Masaryk University, 602 00 Brno, Czech Republic; CEITEC—Central European Institute of Technology, Masaryk University, 625 00 Brno, Czech Republic; National Centre for Biomolecular Research, Faculty of Science, Masaryk University, 625 00 Brno, Czech Republic; Institute of Computer Science, Masaryk University, 602 00 Brno, Czech Republic; CEITEC—Central European Institute of Technology, Masaryk University, 625 00 Brno, Czech Republic; National Centre for Biomolecular Research, Faculty of Science, Masaryk University, 625 00 Brno, Czech Republic; CEITEC—Central European Institute of Technology, Masaryk University, 625 00 Brno, Czech Republic; National Centre for Biomolecular Research, Faculty of Science, Masaryk University, 625 00 Brno, Czech Republic; National Centre for Biomolecular Research, Faculty of Science, Masaryk University, 625 00 Brno, Czech Republic; Department of Physical Chemistry, Faculty of Science, Palacký University Olomouc, 779 00 Olomouc, Czech Republic; CEITEC—Central European Institute of Technology, Masaryk University, 625 00 Brno, Czech Republic; National Centre for Biomolecular Research, Faculty of Science, Masaryk University, 625 00 Brno, Czech Republic

## Abstract

The Protein Data Bank (PDB) is the largest database of experimentally determined protein structures, containing more than 230 000 experimentally determined structures. The chemical reactivity of proteins is based on the electron density distribution, which is usually approximated by partial atomic charges. However, because of the size and high variability, there is not yet a universal and accurate tool for calculating the partial atomic charges of these structures. For this reason, we introduce the web application PDBCharges: a tool for quick calculation of partial atomic charges for protein structures from PDB. The charges are calculated using the recent semi-empirical quantum-mechanical method GFN1-xTB, which reproduces PBE0/TZVP/CM5 charges. The computed partial atomic charges can be downloaded in common data formats or visualized online via the powerful Mol* Viewer. The PDBCharges application is freely available at https://pdbcharges.biodata.ceitec.cz and has no login requirement.

## Graphical Abstract

**Graphical Abstract:**
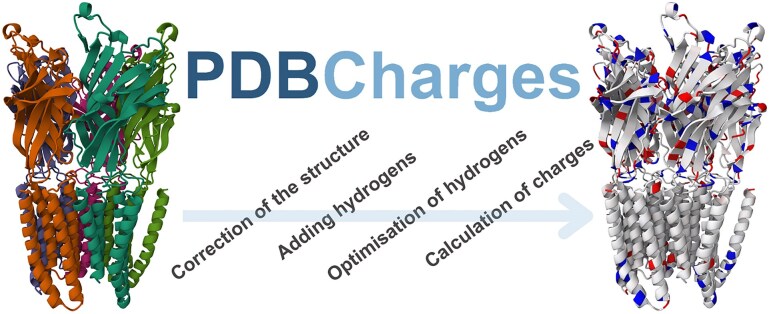

## Introduction

The Protein Data Bank (PDB) [1] is one of the most widely used repositories for three-dimensional macromolecular structural data, including proteins and nucleic acids. It provides access to experimentally determined structures mainly obtained through X-ray crystallography, nuclear magnetic resonance (NMR) spectroscopy, and cryo-electron microscopy. In March 2025, the PDB contains more than 230 000 structures. The structures and information describing their chemical behaviour play a key role for structural biologists and researchers [2, 3]. To meet the demand for these data, the PDBe - Knowledge Base was established to integrate 3D structure data with its functional, biophysical, and biochemical annotations [4].

A typical example of such data is partial atomic charges, which map the electron distribution within the molecule and provide information on its chemical reactivity [5] as the electric fields generated by protein scaffolds are crucial in enzymatic catalysis [6]. Partial atomic charges enable us to compare binding sites, including their polarity [7], help to identify hydrophobic membrane regions [5], or drive protein–protein interactions [8]. These data can be used as input for molecular docking [9] or molecular dynamics [10].

The most reliable method for calculating partial atomic charges is using quantum mechanics (QM) [11], which provides information about electron density distribution among molecular orbitals. Afterwards, its mapping to atoms can be calculated by population analyses, e.g. Natural population analysis [12]. However, QM methods are time-consuming and typically only applicable to small molecules. As a result, standard QM methods cannot be used to calculate the partial atomic charges of structures deposited in the PDB.

A faster alternative is to use empirical charge calculation methods [13], which mimic QM approaches based on significantly simpler empirical equations to describe the charge distribution. However, a drawback of empirical methods is the need for parameterization. Therefore, empirical methods only apply to the types of molecules on which they have been trained [5, 13]. Due to the high variability of, mainly ligand, structures in the PDB, most empirical methods cannot be used to calculate partial atomic charges.

As a solution to this quality versus speed conundrum, a Cover approach has been proposed [5, 14, 15] that significantly accelerates the process while maintaining accuracy. The Cover approach works by dividing the protein structure into smaller segments. The partial atomic charges are then calculated independently for each part. This makes the method scalable enough to be applied to an entire protein structure and its ligands.

Calculating charges for PDB structures is also challenging due to the necessary preprocessing steps. Most structures in PDB do not include hydrogen atoms, which are essential for calculating partial atomic charges. Additionally, many molecular structures deposited in PDB contain various structural issues that must be corrected before calculating partial atomic charges, namely the addition of missing heavy atoms.

For this reason, we introduce the web application PDBCharges. This application offers high-quality partial atomic charges for PDB protein structures in one click. The user has to provide only the PDB ID of the protein, no additional knowledge or preprocessing is required.

## Description of the web server

### Methodology of partial atomic charge calculation

**Figure 1.**
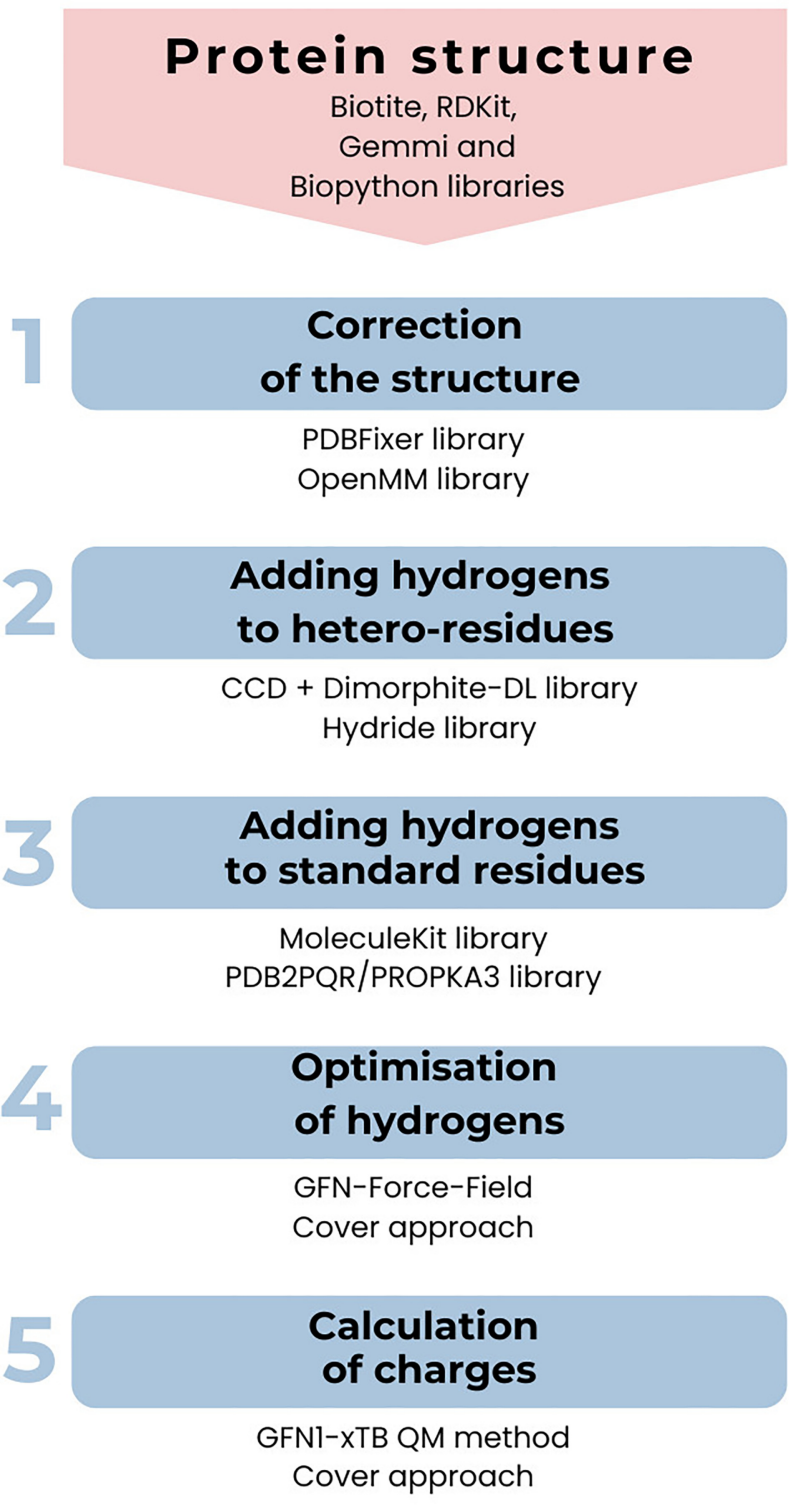
Schematic depiction of PDBCharges methodology of partial atomic charge calculation.

Direct calculation of partial atomic charges for most structures in PDB is impossible due to various issues found in the molecular structures. In addition, most PDB structures are deposited without hydrogen atoms, which are necessary for the calculation of partial atomic charges. To overcome these challenges, the protein structure is first loaded with RDKit [16], Biopython [17], Gemmi [18], and Biotite [19] libraries. Partial atomic charges are then calculated using the methodology illustrated in Fig. 1, which is also described in detail in the user manual available at https://github.com/sb-ncbr/PDBCharges_website/wiki. Methodology for calculating partial atomic charges consists of five steps:

1. *Correction of the structure*: In the first step, the PDBFixer and OpenMM libraries [20] identify and correct structural issues that could degrade the accuracy of partial atomic charge calculations. Specifically, this process involves adding missing heavy atoms to the structure and selecting one position for atoms with multiple alternate positions listed. If the protein structure contains hydrogens, they are removed in this step to ensure consistency in the results.
2. *Adding hydrogens to hetero-residues*: To accurately add hydrogens to hetero-residues, it is necessary first to determine the protonation states of their titratable functional groups. The protonation states are obtained from the Chemical Component Dictionary [21] and then supplied by the Dimorphite-DL library [22]. Once we have the information on the protonation states, the Hydride library [23] assigns the hydrogens to the hetero-residues for physiological pH 7.2.
3. *Adding hydrogens to standard residues*: Protonation states are determined and hydrogens are added to standard residues using the MoleculeKit library [24], which is built on the PDB2PQR/PROPKA3 library [25, 26]. MoleculeKit cannot add hydrogens to hetero-residues. Fortunately, it considers hetero-residues with hydrogens added in the previous step when calculating protonation states. Hydrogens are added for physiological pH 7.2.
4. *Optimization of hydrogens*: The added hydrogens are optimized by the GFN-Force-Field [27]. The calculation of the partial atomic charges is sensitive to the quality of the structure; thus, the optimization of the hydrogen positions improves the accuracy of the resulting charges. Moreover, hydrogens are added to the structure by two different libraries, and the optimization unifies how hydrogen is placed. A cover approach [5, 14, 15] was used to speed up this step.
5. *Calculation of charges*: The calculation of partial atomic charges is performed using the semi-empirical quantum-mechanical method GFN1-xTB [28], which together with the GFN-Force-Field [27] belongs to the family of extended tight-binding quantum chemistry methods whose theory is derived from density functional theory [29]. These methods are available in the xtb package [29]. The GFN1-xTB method is applicable to all atoms in the periodic table up to radon and can, therefore, be used for variable structures such as those in PDB. The GFN1-xTB method reproduces the PBE0/TZVP/CM5 charges [28]. To overcome the issue of computation complexity, PDBCharges uses the cover approach [5, 14, 15]. The calculated charges are then provided to the user via PDBCharges web application.

### PDBCharges web application

PDBCharges web application provides partial atomic charges to protein structures from the PDB database. The user can enter the PDB ID of a selected protein structure on the home page and be redirected to the results page. The user is notified if partial atomic charges cannot be provided for a particular PDB ID (see the *Limitations* section).

The main purpose of the results page is to visualize partial atomic charges and allow users to download these results. The Mol* Viewer [30] visualizes partial atomic charges in several modes for protein structure depending on the selected view with or without water molecules.

In the default *cartoon* representation, each residue is coloured based on the sum of the charges of its atoms. There are two additional visualization modes: *ball*&*stick* and *surface*. The values of charges are represented using a linear blue–white–red gradient, where blue represents a positive charge, white signifies zero charge, and red represents a negative charge. Atoms for which the partial atomic charge calculation could not be carried out are coloured green (see the *Limitations* section).

The application offers two main types of colouring. In the default *relative colouring*, the gradient is mapped to the charges, with the most positive and the most negative charges represented by the most saturated shades of blue and red, respectively. If a user wants to generate multiple images of various structures to compare the charge distribution, *absolute colouring* can be used. It allows manually configuring the maximum values for both positive and negative charges.

The sums of the partial atomic charges of individual residues are generally close to integers. Therefore, it is challenging to distinguish between similarly charged residues. The *highlight charge differences* option mitigates this issue.

While the visual representation of the charges is certainly valuable, users can also download the charges for further processing if required. PDBCharges provides several file formats in a downloaded ZIP file for user convenience. These include:

- PQR file with protein structure and calculated charges (generated by OpenBabel library [31]);
- mmCIF file with protein structure and calculated charges;
- a simple TXT file with calculated charges sorted in atom ordering.

### Limitations

The MoleculeKit library is based on the PDB2PQR/PROPKA3 library, which can only handle structures in PDB format. If a structure is not available in this format, users are notified on the home page that charge calculations cannot be performed.

The charges are calculated only for the first model if multiple models are available for a structure (typically structures determined by NMR). The charges are conformationally dependent and therefore not recommended for molecular dynamics usage directly.

Several issues may occur during the calculation, such as non-convergence of hydrogen optimization or quantum-mechanical charge calculation. However, due to the cover approach, these problems are only local, and the results for the rest of the structure remain unaffected. Users are informed of any problems on the results page. The information about these issues is added to a ZIP bundle with partial atomic charges in a JSON format.

## Results and discussion

We tested the application extensively to confirm that it is stable and reliable. Furthermore, we provide three use cases demonstrating various applications of PDBCharges. They are presented interactively on the PDBCharges webpage.

### Example I: phospholipase inhibited by ibuprofen

**Figure 2.**
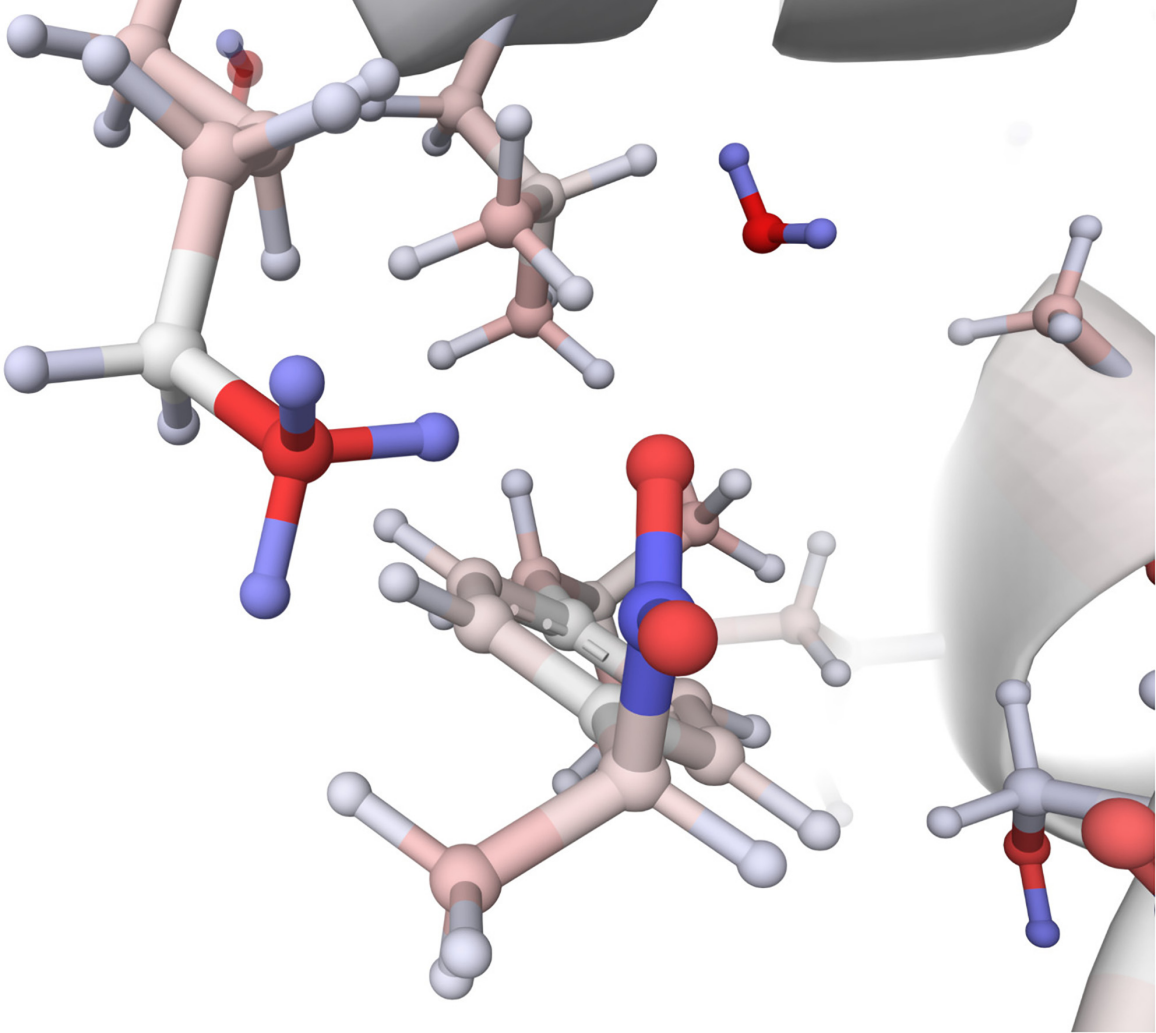
The structure of phospholipase A2 with PDB entry *2pws* with partial atomic charges calculated by PDBCharges is visualized in a ball & stick model that shows the interaction between the negatively charged carboxyl group of ibuprofen and positively charged lysin (LYS 60) in phospholipase A2.

Phospholipase A2 is an enzyme found in plants, mammals, and snake and bee venoms. It catalyses the hydrolysis of the ester bond in phospholipids in the cell membranes. Hydrolysis products are lysophosphatidic acid and free fatty acids, which can disrupt cellular membranes and induce inflammatory responses [32]. The carboxyl group of ibuprofen interacts, creating an electrostatic bond to lysin (LYS 60) in phospholipase A2 (shown in Fig. 2) and inhibiting its function. Based on this knowledge, we can develop therapeutics that can reduce the inflammatory reactions associated with snakebites [33].

### Example II: carbohydrate binding lectin

**Figure 3.**
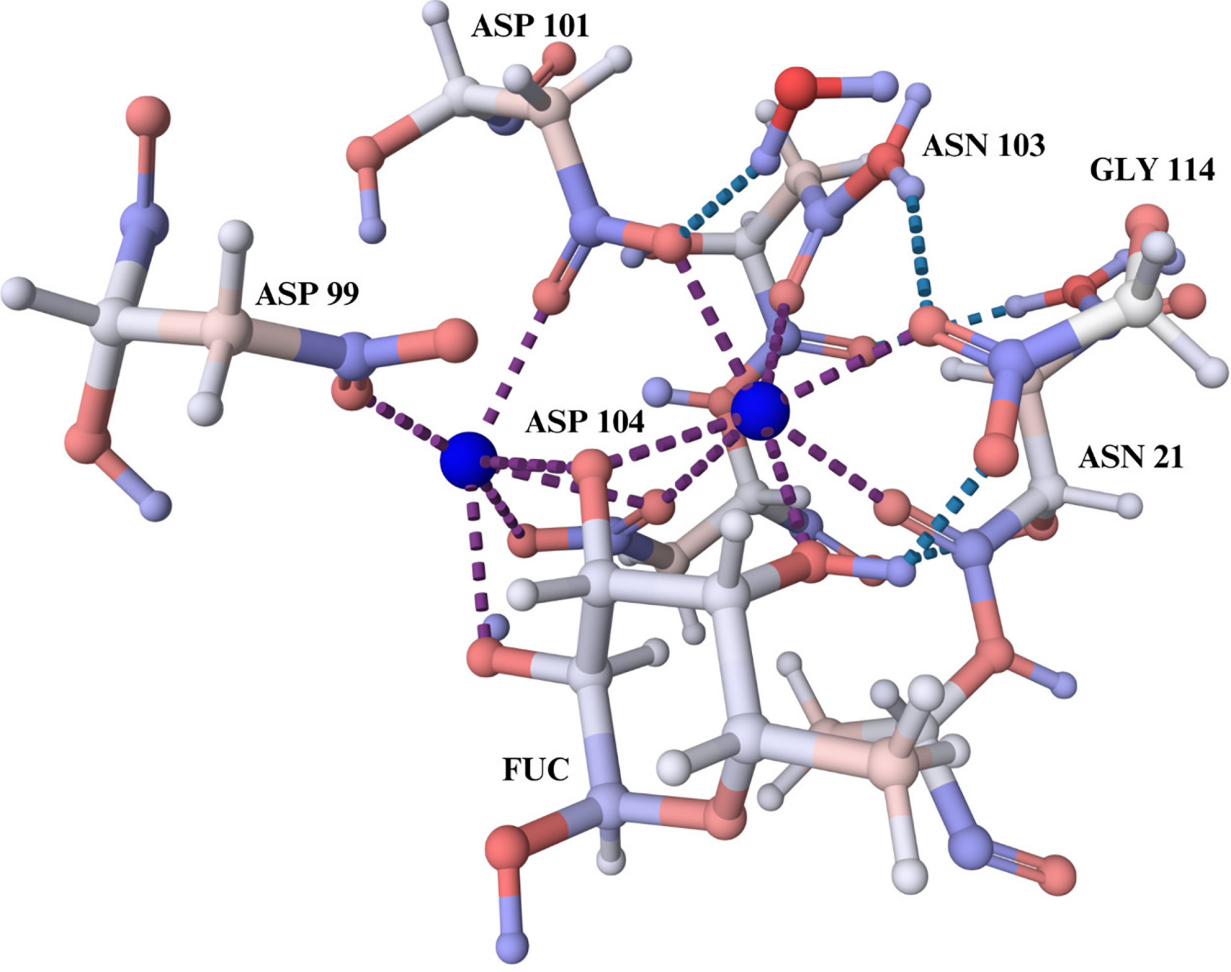
The visualization details the partial atomic charges for the PA-IIL protein structure calculated using PDBCharges. The stable complex depicted is formed by positively charged calcium ions, represented as blue-coloured spheres, interacting with the negatively charged part of residues of the lectin PA-IIL protein (PDB entry *2jdm*) [37].

Lectin PA-IIL from the bacteria *Pseudomonas aeruginosa* plays a key role in its pathogenicity (i.e. it can cause cystic fibrosis, which has high mortality) [34]. This lectin has an unusually high affinity for carbohydrates due to its unique binding mode involving two calcium ions [35]. The calcium ions form ionic bonds with residues in the protein and with three hydroxyl groups of the fucose molecule (chain C: FUC 1118). This interaction (shown in Fig. 3) results in a stable complex through extensive charge delocalization. Unlike most protein–carbohydrate interactions, PA-IIL relies on ionic and coordination bonds with minimal hydrophobic bonds, presenting the structural role of calcium ions in stabilizing the binding site [36, 37].

### Example III: potassium channel

**Figure 4.**
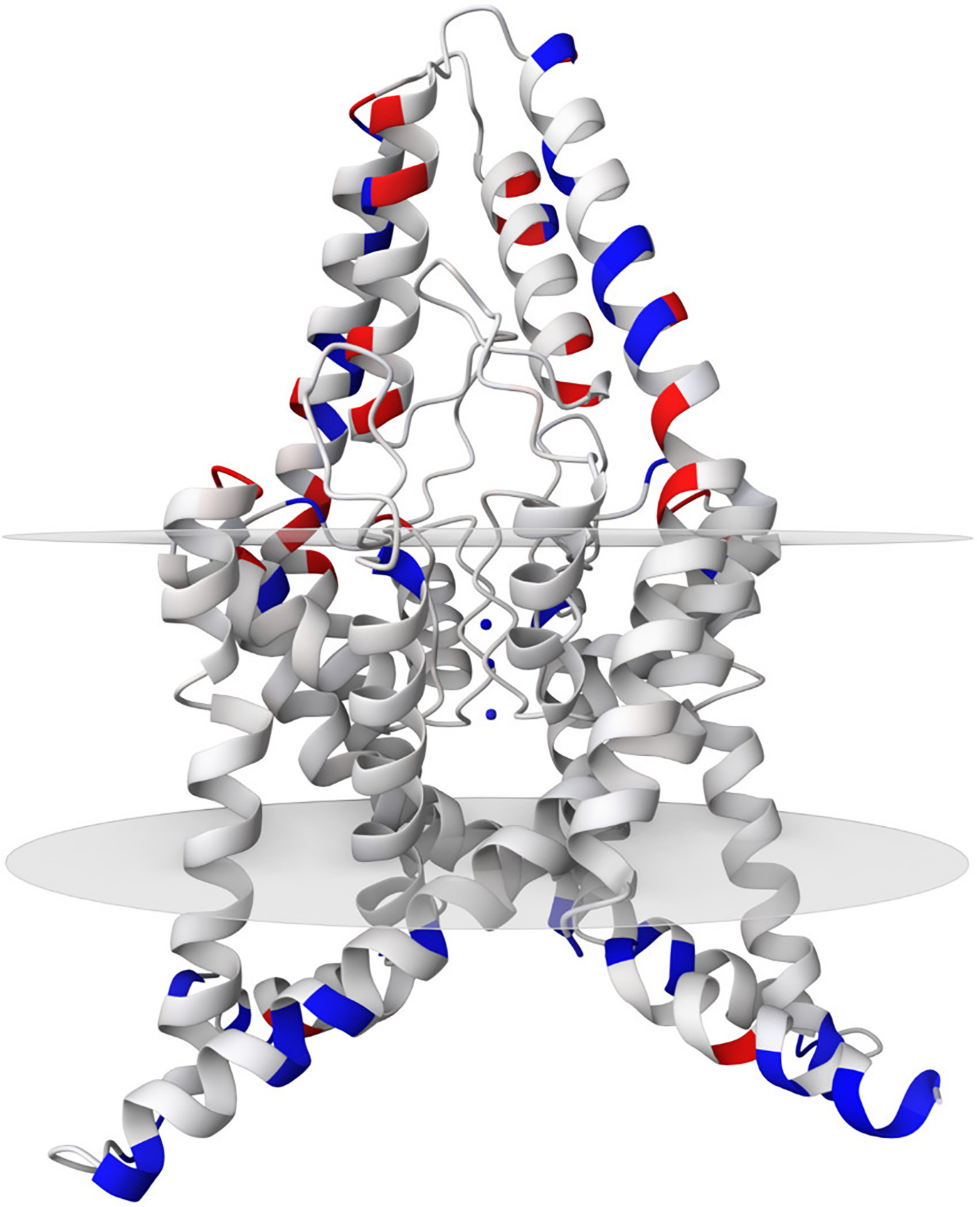
The structure of TASK2 channel from *Mus musculus* with PDB entry *6wlv* [39]. Partial atomic charges, calculated using PDBCharges, are illustrated on a cartoon model to highlight the differences within the protein structure. The sections of the structure located in the intracellular and extracellular regions are polar, with positive charges depicted in red and negative charges in blue. In contrast, the non-polar transmembrane region of the protein is represented in white.

TASK2 (TWIK-related acid-sensitive K^+^ channel 2) is a pH-gated ion channel belonging to the two-pore domain K^+^ channel family [38]. This channel (shown in Fig. 4) maintains cellular homeostasis and regulates physiological responses to environmental changes. The transmembrane regions of TASK2 are characterized by their non-polar nature and lack of charge, distinguishing them from the intracellular and extracellular domains of the protein [39].

## Conclusion

In this article, we presented PDBCharges, a novel web application for calculating partial atomic charges on protein structures available in PDB. PDBCharges adds hydrogens to proteins and their ligands and then utilizes the GFN1-xTB semiempirical quantum-mechanical method, parametrized using PBE0/TZVP/CM5 charges. PDBCharges allows users to download charges (in PQR, mmCIF, or plain text formats) or visualize them via three main structure visualization models (cartoon, ball & stick, and surface). The web application is easy to use and is platform-independent. Documentation explaining the usage of the tool is provided on the webpage.

## Data Availability

PDBCharges application is freely available at https://pdbcharges.biodata.ceitec.cz with no login requirement. The user manual for the application is available at https://github.com/sb-ncbr/PDBCharges_website/wiki. PDBCharges source code is available on GitHub under the MIT licence: the front end and back end are available at https://github.com/sb-ncbr/PDBCharges_website, while the methodology is available at https://github.com/sb-ncbr/PDBCharges. The source code is accessible also at Figshare at https://doi.org/10.6084/m9.figshare.28607612.
